# Integrating RNA-Seq and linkage mapping to identify and characterize *qESCT2*, a cold tolerance QTL at the early seedling stage in rice

**DOI:** 10.3389/fpls.2025.1580022

**Published:** 2025-05-01

**Authors:** Wenqiang Liu, Zuwu Chen, Liang Guo, Zheng Dong, Biaoren Yang, Licheng Liu, Sanxiong Liu, Xiaowu Pan

**Affiliations:** State Key Laboratory of Hybrid Rice, Hunan Hybrid Rice Research Center, Hunan Academy of Agricultural Sciences, Changsha, China

**Keywords:** rice (*Oryza sativa* L.), cold tolerance, RNA-seq, quantitative trait loci (QTL), haplotype, transcription factor

## Abstract

Cold stress significantly limits rice productivity, particularly at the early seedling stage. Identifying key genes responsible for cold tolerance is crucial for breeding resilient rice varieties. In the study, we identified a quantitative trait locus (QTL), q*ESCT2*, associated with cold tolerance at the early seedling stage. The QTL was mapped into an interval of RM1347–RM5356 on chromosome 2 using an F_2:3_ population derived from a cross between XZX45, a cold-sensitive early rice variety from China, and IL43, an introgression line developed by marker-assisted backcrossing. IL43 was created using XN1, a highly cold-resistant cultivar, as the donor parent and XZX45 as the recurrent parent. By integrating transcriptomic data from the target region, we identified *Os02g0181300* as the candidate gene for *qESCT2.* This gene encodes a transcription factor, OsWRKY71. Edited lines of *OsWRKY71* exhibited a significantly lower survival rate under cold tolerance compared to the wild type Nipponbare. Further analysis revealed that *OsWRKY71* likely regulated cold tolerance at the early seedling stage by a glutathione metabolism related pathway. Additionally, *OsWRKY71* exhibits differentiation between *indica* and *japonica* subspecies with distinct haplotypes. These findings will facilitate to further research into the genetic basis of cold tolerance at the early seedling stage and enhance the development of cold-resistant rice varieties by marker-assisted selection.

## Introduction

Rice, a staple food for more than half of the world’s population, is highly sensitive to cold stress due to its tropical and subtropical origins. Cold stress at the early seedling stage has emerged as a significant constraint on rice production. It is estimated that about 7 million hectares of land worldwide are unsuitable for rice cultivation due to cold stress (Liu et al., 2013). Cold damage can affect rice at all developmental stages, but chilling injury at the early seedling stage is particularly detrimental, causing symptoms such as wilting, stunt growth, poor establishment, and ultimately, reduced yields. This issue is particularly critical in double-season rice cultivation areas, where early rice is more vulnerable to cold damage. Therefore, identifying and isolating cold tolerance genes to facilitate breeding cultivars with enhanced cold tolerance at the early seedling stage, especially for early rice in double-season rice areas, remains an urgent priority for improving rice production.

Cold tolerance is a complex quantitative trait controlled by multiple loci. With the rapid development of molecular markers and bioinformatics, significant progress has been made in dissecting complex quantitative traits. To date, a large number of QTLs associated with cold tolerance has been identified (Andaya and Mackill, 2003; Han et al., 2007). Among them, Various populations have been utilized, including F_2_ populations (Liu et al., 2015), recomnbinant inbred line (RIL) populations (Andaya and Mackill, 2003; Jiang et al., 2008; Suh et al., 2012), genome-wide association study (GWAS) populations (Song et al., 2018; Li et al., 2021), doubled haploid (DH) populations (Lou et al., 2007), backcross inbred line (BIL) populations (Fujino et al., 2004; Miura et al., 2002), and near isogenic line (NIL) populations (Saito et al., 1995, 2004, 2010; Park et al., 2013). Numerous genes associated with cold tolerance has been cloned, including *HAN1* (Mao et al., 2019), *COLD1*(chilling-tolerance divergence) (Ma et al., 2015), *CTB1* (cold tolerance at the booting stage) (Saito et al., 2010), *qLTG3-1* (quantitative trait locus for low-temperature germinability) (Fujino et al., 2008), *CTB4a* (Zhang et al., 2017), *COLD11* (Li Z et al., 2023), *LTG1* (low temperature growth) (Lu et al., 2014), *bZIP73* (Liu et al., 2019), *COLD6* (Luo et al., 2024), *CTB3* (Li et al., 2025), *CTB5* (Guo et al., 2025), *CTB6* (Gao et al., 2025), *CTF1*(cold tolerance at the flowering stage) (Dong et al., 2025). However, to date, only few genes for cold tolerance at the early stage were cloned and characterized. While genetic analysis by population construction is a powerful tool for QTL mapping, but it is often time-consuming and labor-intensive. Transcriptomic analysis, on the other hand, offers a more efficient approach by narrowing down candidate genes based on gene expression changes. NIL populations are particularly useful for mapping QTLs with minor effects due to uniform genetic background. Combining NIL population with expression profiling provides a fast and effective strategy for identifying candidate genes. Liu et al. (2013) successfully identified a candidate gene for cold tolerance at the early seedling stage by integrating RNA microarray analysis with an NIL population. This approach highlights the potential of combining genetic and transcriptomic tools to accelerate the discovery of genes underlying complex traits.

In this study, a set of introgression lines (ILs) was developed by marker-assisted backcrossing using Xiangnuo 1 (XN1) as the donor parent and Xiangzhaoxian (XZX45) as the recurrent parent. Among them, IL43, exhibiting high cold tolerance at the early seedling stage, was selected and crossed with the recipient parent XZX45 to construct an F_2:3_ population for mapping QTLs associated with cold tolerance. Furthermore, expression profiles of IL43 and XZX45 were generated. By integrating transcriptomic data with linkage mapping, the candidate gene, *OsWRKY71*, was identified and cloned. Functional validation revealed that it’s edited line significantly decreased cold tolerance at the early seedling stage. This finding offers a valuable genetic resource for enhancing cold tolerance in rice by molecular breeding.

## Materials and methods

### Rice material and population construction

XN1 is a cold-tolerant landrace at the early seedling stage, whereas XZX45 is an early-maturing but cold-sensitive variety. F_1_ plants were generated by crossing XN1, as the donor parent, and XZX45, as the recipient. These F_1_ plants were subsequently backcrossed with XZX45 for four consecutive generations to produce BC_4_F_1_ plants. Additionally, SSR (Simple Sequence Repeat) markers showing clear and polymorphic bands between XN1 and XZX45 were screened. Ultimately, 110 SSR markers evenly distributed across all 12 chromosomes were employed to select ILs from these BC_4_F_1_ plants (**Supplementary Table S1**).

IL43 was crossed with XZX45 to develop a F_2_ population consisting of 171 individual plants. Seeds collected from the F_2_ population were then used to establish the F_2:3_ lines, which were assessed for cold tolerance at the early seedling stage.

### Cold stress scoring at the early seedling stage in rice

Cold treatment was conducted in the growth chamber (LT-361, Percival, USA) following the protocol described in a previous study (Liu et al., 2015). In brief, approximately 60 fully F_3_ seeds were dried at 50°C for 2 days to break dormancy. The seeds were then surface-sterilized and germinated in trays until their coleoptiles reached 5 mm in length. A total of 50 uniformly germinated seeds were selected and subjected to cold stress at 4°C with a relative humidity of 60% for 4 days. After the cold treatments, the seedlings were transferred to a recovery environment with a day/night temperature regime of 28/25°C with a relative humidity of 60% for 7 days. Seedling survival percentage, defined as the ratio of surviving seedlings to the total seedlings exposed to cold treatment, was calculated to evaluate cold tolerance at the early seedling stage. The experiment was arranged in a randomized complete block design with three independent replications. The mean values between replications were used for data analysis, which was performed using DPS software V.2.0 (Tang and Feng, 2007).

### Extraction of DNAs and SSR markers

DNA from the F_2_ population and parental lines was extracted using the CTAB method, as described by Liu et al. (2015). SSR primers were amplified as the previous study (Liu et al., 2015). The resulting PCR products were detected on 6% non-denaturing polyacrylamide gels and visualized via silver staining.

### Construction of linkage map and QTL mapping

Regional linkage maps on chromosomes 2 and 10 were constructed using Mapmaker v.3.0b (Lincoln, 1992). Genetic map distance in centiMorgans (cM) was calculated with the Kosambi function. QTL analysis was conducted using composite interval mapping (CIM) implemented in the Window QTL Cartographer 2.5 software (Wang et al., 2008). The standard CIM model was selected, with forward and backward regression employed for background control. In the CIM analysis, the window size was set to 10 cM (the default value). A threshold of LOD > 2.5 was used to declare the presence of a putative QTL.

### Transicriptome analysis

Germinated seeds with 5 mm bud length from IL43 and XZX45 were subjected to cold stress at 4 °C, buds were harvested at 0, 3 and 12h after stress treatment. Three biological repeats were used for total RNA extraction with TRIzol reagent (Invitrogen). A cDNA library was constructed following Illumina standard protocol and sequenced on a HiSeq 2000 sequencer. All paired-end reads were aligned to the rice Nipponbare reference genome (https://rapdb.dna.affrc.go.jp/download/irgsp1.html). Gene expression levels were calculated using the reads per kilobase per million reads method (RPKM). The differentially expressed genes (DEGs) between IL43 and XZX45 were identified using the criteria of |log_2_^Fold Change^| ≥ 1 and a Q ≤0.05.

A hypergeometric test was applied (P ≤ 0.05) to identify significantly enriched Gene Ontology (GO) terms by comparing DEGs to the genomic background. Additionally, KEGG pathway analysis was conducted using the blastall program against the KEGG database (http://www.genome.jp/kegg). Pathway enrichment analysis was performed to identify significantly enriched metabolic and signal transduction pathways associated with DEGs (P ≤ 0.05).

### Plasmid construct and transformation

To construct the clustered regularly inter-spaced short palindromic repeats (CRISPR)/CRISPR-associated nuclease 9 (Cas9) vector, single guide RNAs (sgRNAs) targeting the coding regions of *Os02g0181300* and *Os02g0207400* were designed. The expression cassettes, OsU3-*Os02g0181300*-sgRNA and OsU3-Os02g0207400-sgRNA, were subsequently assembled to create two new vectors, pYL-HU-U3-Cr*Os02g0181300* and pYL-HU-U3-CrOs*02g0207400*. These constructs were introduced into Nipponbare mature embryo-induced calli by *Agrobacterium*-mediated transformation (EHA105). The primers used are listed in **Supplementary Table S2**.

### Allelic variation and haplotype analysis

The primers were designed for amplifying coding regions of *Os02g0181300* from IL43 and XZX45. Haplotype network analysis was used from the website (http://ricevarmap.ncpgr.cn/). The primers used are listed in **Supplementary Table S2**.

## Results

### Development and identification of ILs

**Figure 1:**
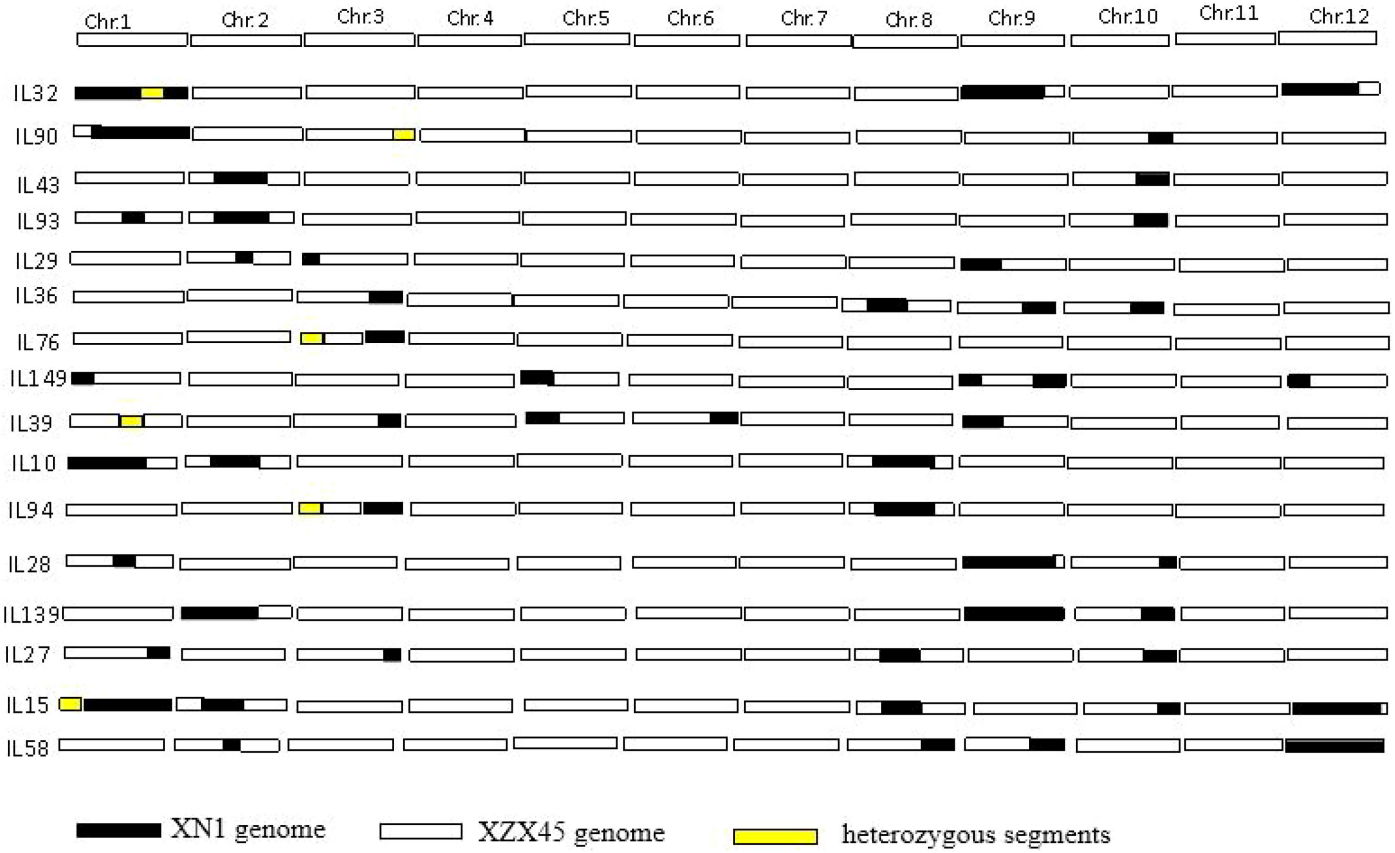
Selected ILs by marker-assisted backcross derived from XN1 as donor parent and XZX45 as recurrent parent. XN1, Xiangnuo 1. XZX45, Xiangzaoxian45.

A total of 16 ILs were selected on the basis of SSR marker genotypes, collectively covering approximately one-third of the donor genome. Among these, chromosomes 1, 9, and 12 of the donor genome were almost entirely represented. Each IL contained more than two donor chromosomal segments, ranging in size from approximately 2.1 to 27.3 Mb. Specifically, IL43 carried two donor segments: one in the interval of RM1347–RM341 on chromosome 2 (14.0 Mb) and another in the interval of RM271–RM228 on chromosome 10 (5.6 Mb) (**Figure 1**).

**Figure 2:**
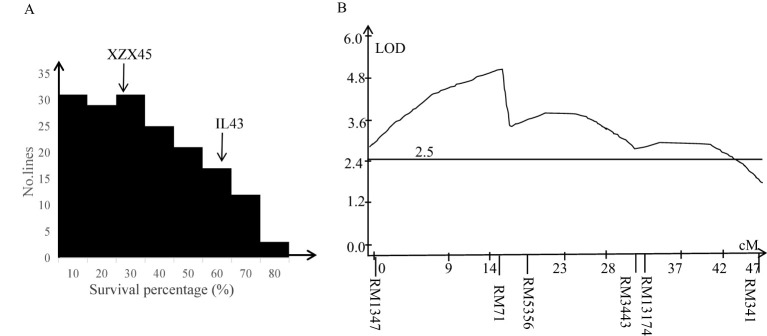
Frequency distribution of survival percentage **(A)** and a curve line diagram for QTL mapping **(B)** in an F_2:3_ population derived from the cross between XZX45 and IL43.

Cold tolerance evaluation between IL43 and XZX45 revealed that IL43 exhibited significantly higher cold tolerance at the early seedling stage, with a survival rate of 63.5%, compared to 32.6% for XZX45 (**Figure 2A**). These results suggest that IL43 likely harbors QTLs associated with cold tolerance, potentially derived from the donor genome.

### Delimitation of confidence interval of QTL for cold tolerance

To dissect the QTL associated with cold tolerance at the early seedling stage, an F_2_ population derived from XZX45/IL43 cross was developed. Two regional linkage maps were constructed on chromosome 2 and 10 in the F_2_ population, spanning 46.8 cM and 37.5 cM, respectively. The frequency distribution of seedling survival percentage in the F_2:3_ lines showed continuous variation (**Figure 2A**), suggesting that the QTL primarily played an additive effect.

QTL analysis identified a significant QTL with peak located in the interval of RM1347–RM5356 on chromosome 2. This QTL had a LOD score of 5.15 and accounted for 29.51% of the phenotypic variation (**Figure 2B**). The donor-derived allele at this locus was found to enhance cold tolerance in the population. In contrast, no significant QTL was detected in the interval of RM271–RM228 on chromosome 10.

### DEGs identified in the target region

**Figure 3:**
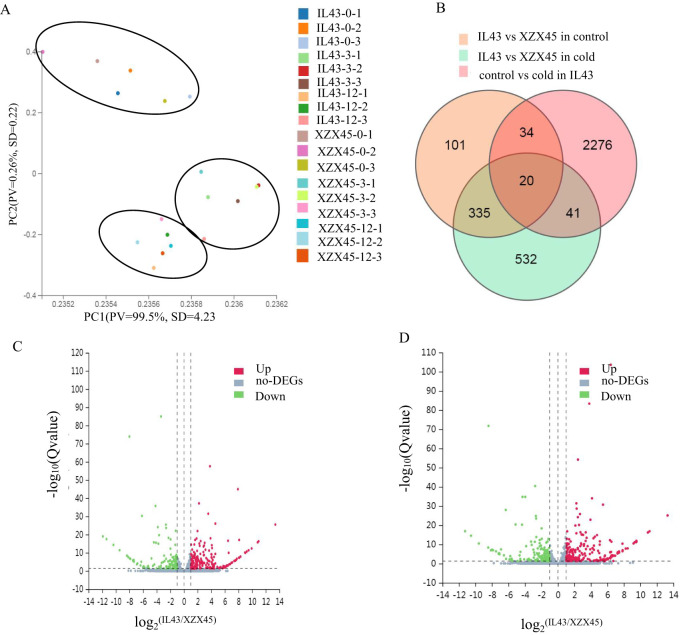
Principal component analysis (PCA), identification of differentially expressed genes (DEGs), and volcano plots. **(A)** Distribution of XZX45 and IL43 along the first two principal components (PC1 and PC2). **(B)** Venn diagram showing DEGs between XZX45 and IL43 under different conditions. **(C)** Volcano plot comparing XZX45 with IL43 under normal temperatures. **(D)** Volcano plot comparing XZX45 with IL43 under low temperatures.

In order to identify candidate genes within the interval of RM1347–RM5356, genome-wide gene expression profiling of IL43 and XZX45 was investigated at 0, 3 and 12h after cold stress. Principal component analysis (PCA) of the RNA-sequencing (RNA-seq) data revealed that principal components 1 accounted for 99.5% of the total variability. The PCA results showed three distinct clusters, with each cluster containing samples from IL43 and XZX45 corresponding to the same time point (**Figure 3A**). This clustering pattern suggests that IL43 and XZX45 had high expression similarity. The result further supported that IL43 and XZX45 share nearly identical genetic backgrounds.

A total of 490 and 928 DEGs were identified under normal conditions and cold stress, respectively. Under normal condition, 284 genes were up-regulated and 206 genes were down-regulated (**Figure 3B**). Under cold stress, 401 genes were up-regulated and 527 genes were down-regulated (**Figure 3C**). The relatively small number of DEGs under both conditions further supports the conclusion that IL43 and XZX45 share nearly identical genetic backgrounds. This finding is consistent with the SSR marker detection results, which exhibited that IL43 carries only two short donor segments.

### Analysis of candidate genes

A Venn diagram comparing gene expression profiles among IL43 vs XZX45 under control conditions, IL43 vs XZX45 under cold stress, and IL43 under both control and cold stress revealed 20 genes with significant expression changes (**Figure 3D**). When combined with the QTL mapping region, four genes were identified within the RM1347–RM5356 interval. Among them, two genes (*Os02g0181300* and *Os02g0207400*) exhibited relatively high expression fold changes across different treatment time points, with *Os02g0181300* being up-regulated and *Os02g0207400* down-regulated (**Table 1**). In order to further validate these findings, RT-qPCR (real time quantitative polymerase chain reaction) was performed for *Os02g0181300* and *Os02g0207400*. The RT-qPCR results revealed expression patterns consistent with the RNA-seq data between IL43 and XZX45 (**Supplementary Figure S1**). Consequently, *Os02g0181300* and *Os02g0207400* were selected as candidate genes based on its higher expression fold change.

**Table 1:** Fold changes of differentially expressed genes (DEGs) in the interval of RM1347–RM5356 between IL43 and XZX45.

Accesssion	Anotation	0h	3h	12h
		Log_2_^(IL43/XZX45)^	Log_2_^(IL43/XZX45)^	Log_2_^(IL43/XZX45)^
Os02g0181300	WRKY71	0.65	1.38	2.78
Os02g0207400	cytokinin-O-glucosyltransferase 3	-0.41	-2.43	-3.22
Os02g0215000	nuclear protein ZAP-related	1.07	0.94	0.97
Os02g0222500	expressed protein	1.41	1.30	1.05

### Functional validation of *Os02g0181300* in cold tolerance

**Figure 4:**
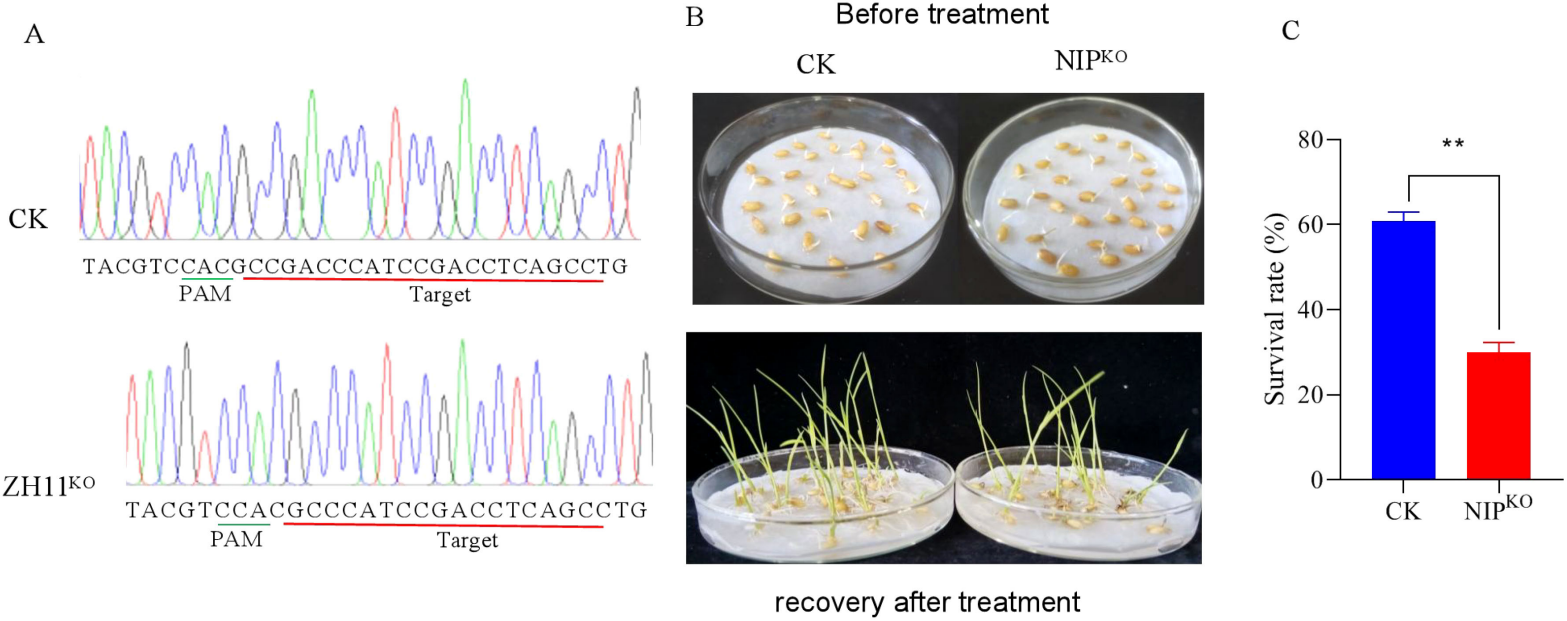
CRISPR/Cas9 knockout of *Os02g0181300* (NIP^KO^) in rice. **(A)** Targeted mutation in *Os02g0181300*. **(B)** Phenotypic comparison of wild-type and NIP^KO^ after cold treatment at 4°C for 5 days followed by a 7-day recovery. **(C)** Seedling survival rates of NIP^KO^ and wild-type plants. ** significant difference at P <0.01 level.

In order to further determine which of *Os02g0181300* and *Os02g0207400* is responsible for cold tolerance at the early seedling stage, each gene was separately knocked out in the Japonica rice variety Nipponbare (NIP) using Crisp/Cas9 technology. We obtained five transgenic lines for *Os02g0181300* and four for *Os02g0207400.* Sequencing of the edited lines identified a homozygous line (NIP^KO^) with a 4-bp deletion in *Os02g0181300* (**Figure 4A**). Evaluation of cold tolerance showed that the seedling survival rate of NIP^KO^ was significantly lower than that of the wild-type following low-temperature treatment (**Figures 4B, C**). Besides, the homozygous seeds for the edited *Os02g0207400* were not available from the segregating progeny of the four heterozgyous transgenic lines, we speculated that seeds could not survive in the absence of *Os02g0207400*. These results demonstrate that *Os02g0181300* plays a critical role in cold tolerance at the early seedling stage. *Os02g0181300* encoded a WRKY transcription factor and has been annotated as *OsWRKY71. OsWRKY71* was reported to be involved in rice defense response and cold-responsive at the seedling stage (Liu et al., 2007; Kim et al., 2016).

### Allelic variation and haplotype analysis of *OsWRKY71*

**Figure 5:**
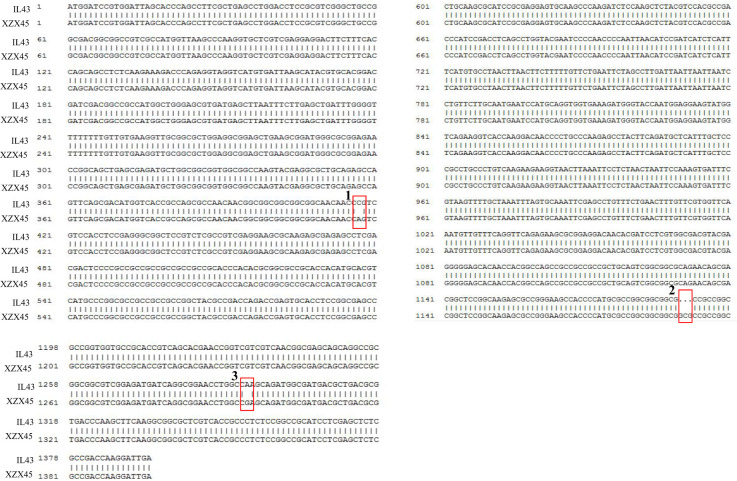
Sequence alignment of the coding region of *Os02g0181300* between IL43 and XZX45. In red rectangular box 1, the nucleotide has a substitution from C in IL43 to A in XZX45, resulting in an amino acid change from proline (P) to glutamine (Q). In red rectangular box 2, a 3-bp deletion in IL43 result in an amino acid deletion of alanine (A). In red rectangular box 3, the nucleotide has a substitution from C in IL43 to A in XZX45, resulting in an amino acid change from lysine (K) to glutamic acid (E).

To further investigate the potential role of *OsWRKY71* in cold tolerance, the alleles from IL43 and XZX45 were cloned and sequenced. Sequence comparison revealed two single nucleotide polymorphisms (SNPs) and one 3-bp insertion/deletion (INDEL) in the coding regions of *OsWRKY71*. These variations resulted in two amino acid changes and one amino acid deletion in IL43 (**Figure 5**), suggesting that these variations likely contributed to the varying level of cold tolerance.

**Figure 6:**
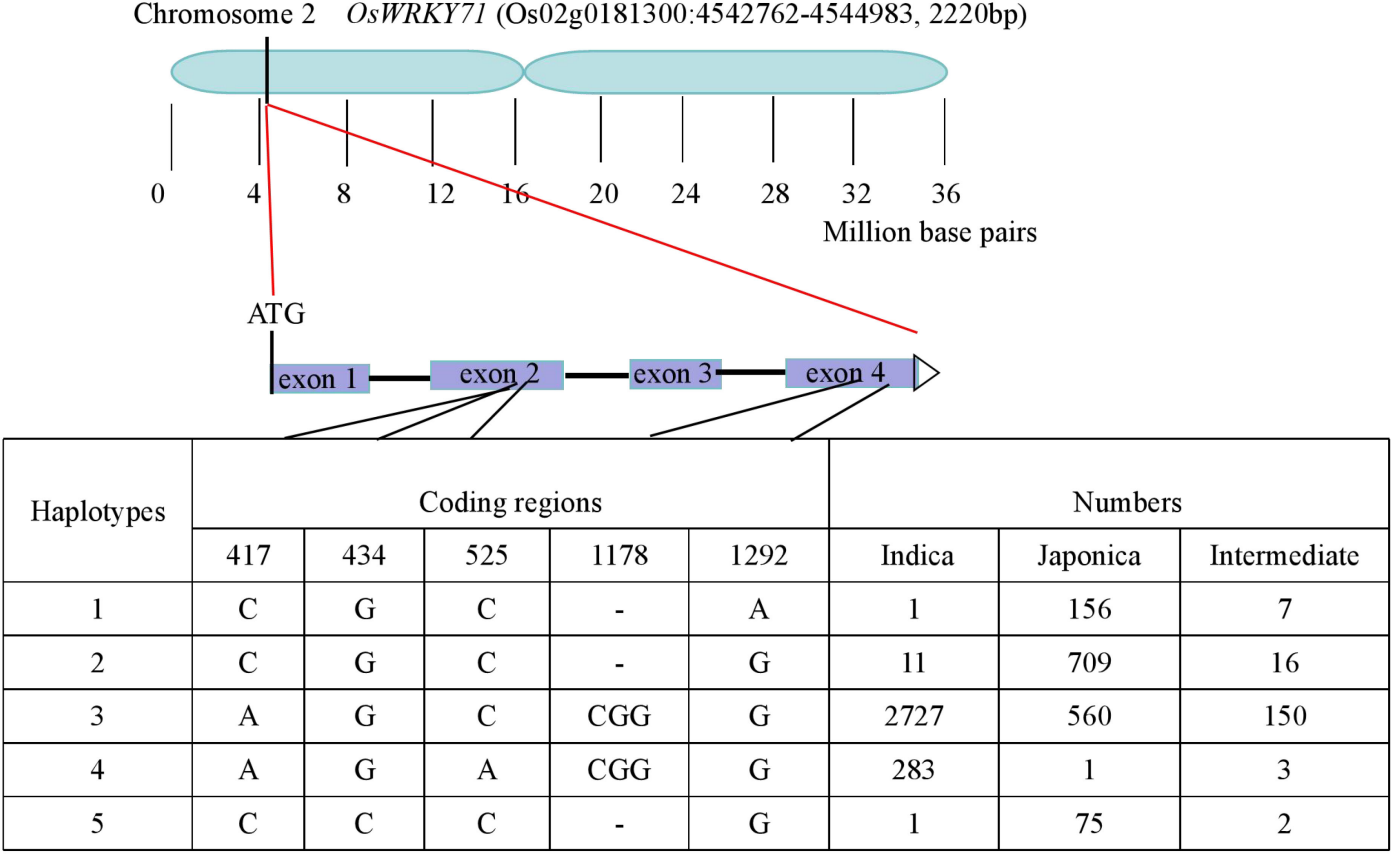
Haplotype analysis of *OsWRKY71* according to the data from the website (http://ricevarmap.ncpgr.cn/).

To further clarify the haplotypes of *OsWRKY71*, natural variations in the coding region of the gene was subsequently investigated from the website (http://ricevarmap.ncpgr.cn/). As a result, a total of five sites with amino acid changes were identified among 4696 rice accessions. Based on these polymorphisms, sequences of these accessions were categorized into five haplotypes. Among these, Hap1,Hap2 and Hap5 were predominantly presented in *japonica* cultivar, while Hap4 was almostly exclusively found in *indica* cultivar. Notably, Hap3 was observed in both *indica* and *japonica* cultivar, representing approximately half of total rice accessions (**Figure 6**). Hap3 appeared to be undergoing differentiation toward *indica* and *japonica* subspecies. The IL43 allele was found to belong to Hap1, while the XZX45 allele was classified as Hap3. These findings suggest that IL43 allele could be utilized to improve cold tolerance at the early seedling stage in rice.

### Regulatory network underlying *OsWRKY71*-mediated cold tolerance

**Figure 7:**
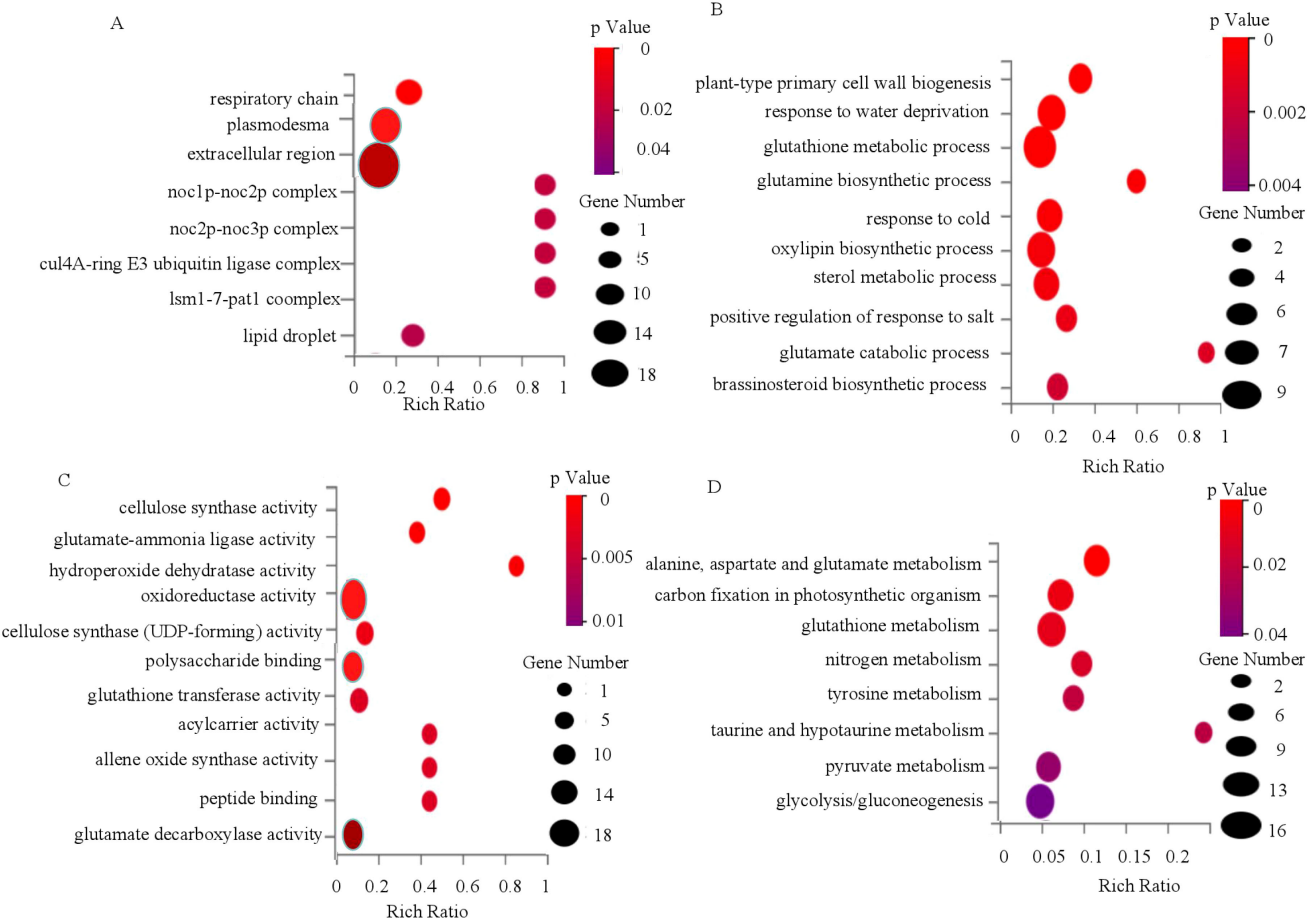
GO and KEGG enrichment analysis of DEGs between XZX45 and IL43 under cold stress. **(A)** Enrichment analysis of DEGs in the cellular component category. **(B)** Enrichment analysis of DEGs in the biological process category. **(C)** Enrichment analysis of DEGs in the molecular function category. **(D)** KEGG enrichment analysis of DEGs between XZX45 and IL43.

To elucidate the regulatory network underlying *OsWRKY71*-mediated cold tolerance, we conducted transcriptome analysis to identify DEGs between IL43 and XZX45 after cold treatment. GO enrichment analysis of these DEGs revealed significant terms in the cellular component category, including “respiratory chain,” “plasmodesma,” “extracellular region,” “Noc1p-Noc2p complex,” and “Noc2p-Noc3p complex” (**Figure 7A**). In the biological process category, enriched terms encompassed “plant-type primary cell wall biogenesis,” “response to water deprivation,” “glutathione metabolic process,” “glutamine biosynthetic process,” and “response to cold” (**Figure 7B**). For molecular function, enriched terms included “cellulose synthase activity,” “glutamate-ammonia ligase activity,” “hydroperoxide dehydratase activity,” “oxidoreductase activity,” and “cellulose synthase (UDP-forming) activity” (**Figure 7C**). These findings suggest that *OsWRKY71* may regulate cellulose synthase activity and modulate glutamine and glutathione metabolism in rice. To further explore pathways associated with *OsWRKY71*, we mapped the DEGs in the KEGG database and performed pathway enrichment analysis. The top five enriched pathways were “Alanine, aspartate, and glutamate metabolism,” “Carbon fixation in photosynthetic organisms,” “Glutathione metabolism,” “Nitrogen metabolism,” and “Tyrosine metabolism” (**Figure 7D**). Therefore, we speculated that glutathione metabolism might involved in cold tolerance pathway.

**Figure 8:**
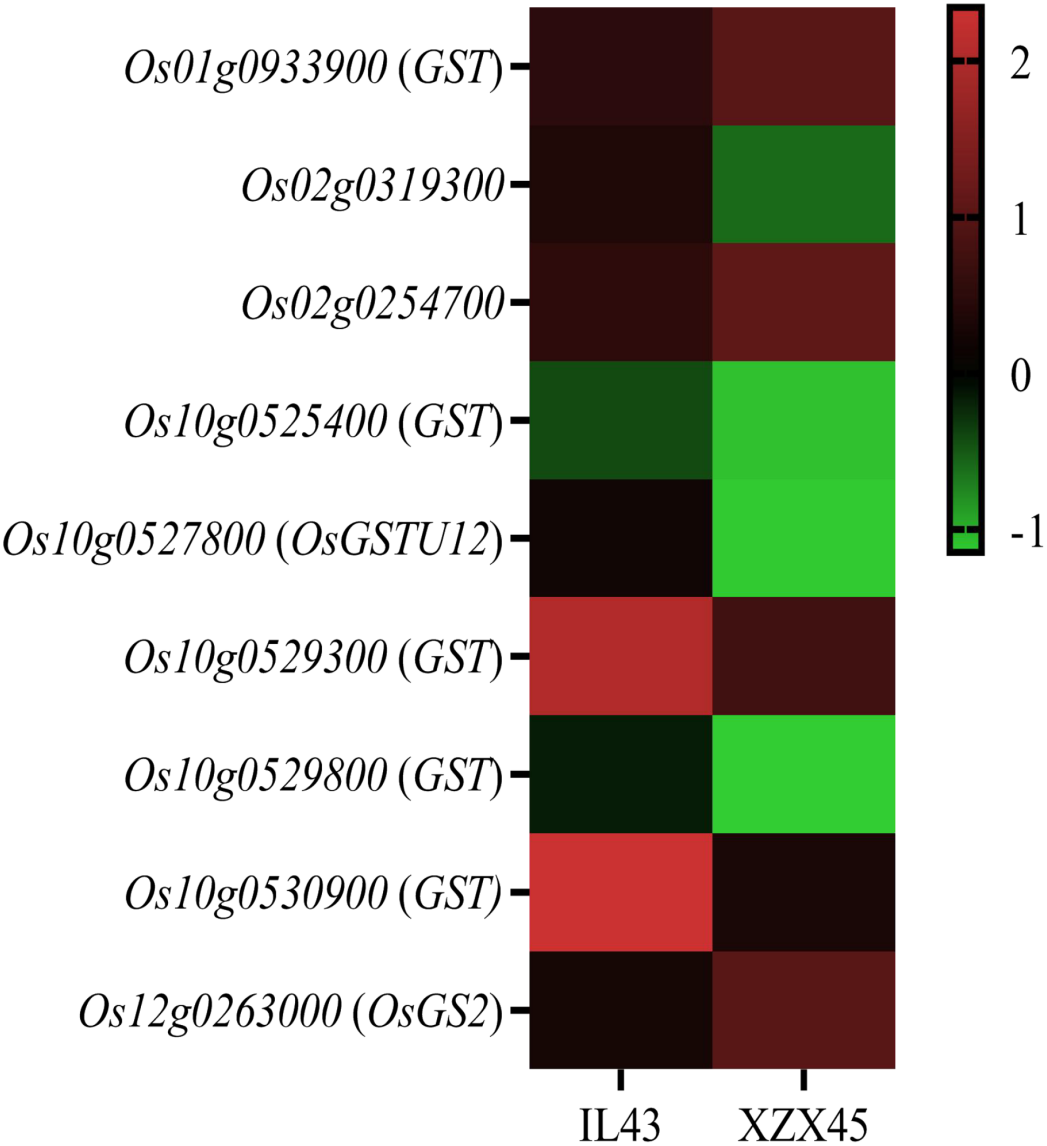
Heat map of glutathione metabolism related genes from buds subject to cold tolerance at 4°C between IL43 and ZXZ45. GST glutathione S-transferase.

To further validate it, some gultathione-related genes, such as encoded glutathione S-transferase and hydroxymethyl-glutathione synthetase, were investigated for their expression level in IL43 and XZX45 after cold treatment. The results revealed that these genes showed significantly differential expression (**Figure 8**). Collectively, the transcriptomic data suggests a potential link between *OsWRKY71* and glutathione metabolism, but further biochemical validation is required.

## Discussion

Genetic identification of quantitative trait loci (QTL) associated with complex traits often involves delimiting QTL into small intervals using molecular markers. The next challenge is determining the target genes within these intervals. RT-qPCR is commonly used to analyze the expression levels of genes within these regions to identify candidate genes. With the advent of high-throughput expression profiling and the construction of RNA-Seq libraries for rice exposed to various stress conditions—such as cold and salt stress—it has become increasingly feasible to identify stress-induced genes within target regions (Liu et al., 2013). When comparing two parents with contrasting genetic backgrounds, a large number of DEGs are typically observed, which can help elucidate the overall mechanism underlying the trait of interest. However, using a pair of NILs that share nearly identical genetic backgrounds, except for a few donor segments, makes it easier to pinpoint candidate genes within target regions based on changes in expression levels. In this study, IL43, an introgression line, was developed through marker-assisted backcrossing using XN1 as the donor parent and XZX45 as the recurrent parent. Genome-wide detection with SSR markers confirmed that IL43 carried only two donor segments. Compared to XZX45, IL43 exhibited significantly higher cold tolerance at the early seedling stage. As expected, a QTL associated with cold tolerance at this stage was detected in the interval of RM1347–RM5356 in the donor segment, using an F_2:3_ population derived from the cross between IL43 and XZX45. The allele from the donor parent increased cold tolerance. By integrating transcriptomic data, only four genes within the RM1347–RM5356 interval showed differential expression. Among these, *OsWRKY71* was validated to be responsible for cold tolerance at the early seedling stage. This study demonstrates that combining RNA-Seq with linkage mapping not only accelerate the identification of candidate genes but also provide a more efficient and precise method for dissecting complex quantitative traits.

Cold tolerance in plant is a complex regulatory network involving multiple genes and pathways. These genes interact in intricate ways to help plants sense, respond to, and survive under cold stress. Currently, most studies have focused on the ICEs (inducer of CBF expression)-CBF/DREB1s (C-repeat binding factor/dehydration-responsive element binding protein 1)-CORs (cold-regulated) signaling pathway (Chinnusamy et al., 2007; Zarka et al., 2003). In this pathway, ICE proteins directly bind the promoters of *CBF*/*DREB1* genes to positively regulate their expression. Subsequently, CBF/DREB1 proteins activate the expression of COR genes, triggering a signaling network that enhances cold tolerance (Guo et al., 2018). In addition to the ICE-CBF/DREB1-COR pathway, other transcription factors have been reported to play roles in cold tolerance. The bZIP73^Jap^ interacts with bZIP71 to regulate the expression of *qLTG3-1^NIP^*, overexpression lines of *qLTG3-1^Nip^* greatly improved rice tolerance to cold stress (Liu et al., 2019). CTB5, a homeodomain-leucine zipper (HD-Zip) transcription factor, interacts with OsHox12 and targets gibberellin (GA) metabolism genes to promote GA accumulation in anthers, facilitating tapetum development under cold stress (Guo et al., 2025). CTB3, a calmodulin-binding transcriptional activator, activates the expression of trehalose-6-phosphate phosophatase1 (*OsTPP1*), reducing trehalose 6-phosphate (*Tre6P*) levels. This leads to increase sugar accumulation in panicles and improved cold tolerance. In this study, *Os02g0181300* encodes a WRKY71 transcription factor, a member of the WRKY family. Rice *WRKY* genes are known to be involved in phytohormone signaling pathways, including abscisic acid (ABA) (Xie et al., 2005) and GA (Zhang et al., 2004). Some *WRKY* genes, such as *OsWRKY45* (Tao et al., 2011), *OsWRKY71* (Kim et al., 2016) and *OsWRKY24* (Li J. et al., 2023), showed responsive expression in response to cold stress. Although previous studies have demonstrated that *OsWRKY71* was responsive to cold stress at the seedling stage (Kim et al., 2016), more validation is lack. Here, *OsWRKY71* was identified to be responsible for cold tolerance at the early seedling stage. These results indicate that *OsWRKY71* likely plays a role both at the early seedling and at the seedling stage. However, the mechanisms by which *OsWRKY71* regulates cold tolerance, including the potential involvement of ABA or GA signaling pathways, remain to be further elucidated.

When plants are exposed to cold stress, cellular homeostasis can be disrupted, leading to the overproduction of reactive oxygen species (ROS). In plants, ROS are highly toxic at elevated concentrations and must be tightly regulated through a balance between production and scavenging systems (Qi et al., 2017). To counteract oxidative damage, plants have evolved a suite of scavenging enzymes, including ascorbate peroxidase, superoxide dismutase, catalase, and glutathione peroxidase (Foyer and Noctor, 2005). Glutathione, a tripeptide composed of glutamic acid, cysteine, and glycine, plays a central role in plant responses to abiotic stresses, including cold tolerance. As a key component of the cellular antioxidant system, glutathione helps mitigate the harmful effects of ROS generated under cold stress. In this study, glutathione and glutathione-related metabolic pathways were frequently enriched in both KEGG and GO analyses. These findings suggest that glutathione and its associated metabolism may play a critical detoxifying role in defending against chilling stress. However, the involvement of glutathione-related genes in cold tolerance regulation still requires further verification.

Owing to the long-term domestication and natural selection process, during which *indica* and *japonica* rice have adapted to distinct ecological environments and climatic conditions, most cold-tolerance genes display significant differentiation between the *indica* and *japonica* subspecies. For example, genes such as *HAN1* (Mao et al., 2019), *bZIP73* (Liu et al., 2019), *COLD1* (Ma et al., 2015), and *CTB4a* (Zhang et al., 2017) exhibit favorable haplotypes that are specifically present in *japonica* rice and significantly enhance cold tolerance. Like other cold-tolerance genes, *OsWRKY71* exhibits clear differentiation between *indica* and *japonica* subspecies in this paper. The favorable haplotye (Hap1) was present in cold-tolerant *japonica* rice and showed enhanced cold tolerance at the early seedling stage. This finding offers a valuable genetic resource for enhancing cold tolerance of early-season rice varieties.

## Data Availability

Publicly available datasets were analyzed in this study. This data can be found here: https://rapdb.dna.affrc.go.jp/download/irgsp1.html.
