# Supplementary material for: Integrating RNA-Seq and linkage mapping to identify and characterize qESCT2, a cold tolerance QTL at the early seedling stage in rice
Source: Front Plant Sci. 2025 May 1;16:1580022. doi: 10.3389/fpls.2025.1580022 (PMC12078224; doi:10.3389/fpls.2025.1580022)
Supplement: Supplementary file 1 [file DataSheet1.doc]

Integrating RNA-Seq and linkage mapping to identify and characterize qESCT2, a cold tolerance QTL at the early seedling stage in rice

Wenqiang Liu, Zuwu Chen, Liang Guo, Zheng Dong, Biaoren yang, Licheng Liu, Sanxiong Liu, Xiaowu Pan^*^

(State Key Laboratory of Hybrid Rice, Hunan Hybrid Rice Research Center, Hunan Academy of Agricultural Sciences, Changsha 410125, China)

Supplementary Table S1 SSR marker names used for marker-assisted selection on 12 chromosomes

| Chromosome | SSR marker names |
| --- | --- |
| 1 | RM3426 RM259 RM243 RM1032 RM23 RM10828 RM6771 RM466 RM6880 RM11383 RM246 RM128 RM265 RM472 RM104 |
| 2 | RM211 RM233 RM1347 RM71 RM5356 RM3443 RM13174 RM341 RM5427 RM450 RM240 RM166 |
| 3 | RM3413 RM6301 RM14586 RM218 RM3434 RM15090 RM7431 RM16 RM8209 RM520 RM143 RM148 |
| 4 | RM451 RM7200 RM5953 RM16686 RM17054 RM252 RM470 RM348 |
| 5 | RM13 RM3777 RM289 RM249 RM6229 RM305 RM5970 RM3170 |
| 6 | RM508 RM6119 RM3431 RM3183 RM4924 RM275 RM5371 RM3307 |
| 7 | RM5752 RM7121 RM3859 RM214 RM500 RM11 RM10 RM234 RM6063 RM172 |
| 8 | RM407 RM38 RM72 RM331 RM339 RM7556 RM477 |
| 9 | RM285 RM5799 RM5526 RM24181 RM566 RM288 RM7306 |
| 10 | RM222 RM216 RM3311 RM5689 RM271 RM147 RM228 |
| 11 | RM7557 RM536 RM6091 RM206 RM6499 RM224 |
| 12 | RM247 RM7119 RM27902 RM7102 RM1261 RM3331 RM6947 RM235 |

Supplementary Table S2 List of primers used in the study

| Primer | Sequences (5'-3') | Experiments |
| --- | --- | --- |
| qRT2g0181300F | TCCGTGGATTAGCACCCAGC | RT-qPCR |
| qRT2g0181300R | GCTGCTGGTGAAAGAAGTCCT | RT-qPCR |
| qRT2g0207400F | CGTCGAGAACGTCGACCAG | RT-qPCR |
| qRT2g0207400R | CGCGCAGGTACGCCTC | RT-qPCR |
| Actin F | tggcatctctcagcacattcc | RT-qPCR |
| Actin R | tgcacaatggatgggtcaga | RT-qPCR |
| CDS2g0181300F | CCTCCCTCCTCCCATGACTA | Sequence alignment |
| CDS2g0181300R | CGCGTCGATCGTGTTCTTG | Sequence alignment |
| Cr2g0181300F | cagtggtctcCCACGCCGACCCATCCGACCTCA | Vector construction |
| Cr2g0181300R | cagtggtctcTGAGGTCGGATGGGTCGGCGTGG | Vector construction |
| Cr2g0207400F | cagtggtctcGCCCGACGTCGCCCAGAGCGTGG | Vector construction |
| Cr2g0207400R | cagtggtctcCCACGCTCTGGGCGACGTCGGGC | Vector construction |

Supplementary Figure S1 Relative expression level of *Os02g0181300* and *Os02g0207400* after cold treatment at 0, 3, 12 h between IL43 and XZX45. * significant difference at P <0.05 level, ** significant difference at P <0.01 level
